# Explainable Quality Assessment and Measurement from Real-World Hip Ultrasound Cine Sweeps

**DOI:** 10.3390/bioengineering13060667

**Published:** 2026-06-08

**Authors:** Adam McArthur, Stephanie Wichuk, Stephen Burnside, George Reed, Sukhdeep Dulai, Abhilash Hareendranathan, Jacob L. Jaremko

**Affiliations:** 1 Department of Radiology and Diagnostic Imaging, University of Alberta, Edmonton, AB T6G 2R3, Canada; 2 Faculty of Medicine, University of Alberta, Edmonton, AB T6G 2R3, Canada

**Keywords:** developmental dysplasia of the hip, hip ultrasound, cine sweep ultrasound, deep learning, explainable AI, medical imaging, biomedical applications

## Abstract

This study evaluates Retuve, an open-source explainable pipeline for the automated analysis of infant hip ultrasound cine sweeps. Retuve combines segmentation, Graf-plane calibration, and frame filtering. In a retrospective multicenter study, we tested the full pipeline on an external set of 109 hips from a Canadian community clinic, with internal developmental validation of segmentation on 90 hips and Graf-plane calibration on 419 hips. On the external test set, Retuve achieved 100% specificity and 91% sensitivity for expert agreement regarding whether a sweep contained an analyzable frame, compared with 75% specificity and 96% sensitivity for a radiology fellow; specificity was based on 16 expert-negative examinations. For alpha angle and acetabular coverage, Retuve achieved consistency intraclass correlation coefficients (ICCs) of 0.77 and 0.74, comparable to the fellow’s 0.70 and 0.74. However, alpha-angle absolute agreement was lower (ICC 0.55, 95% confidence interval (CI) −0.07–0.81), consistent with systematic measurement bias. Internal developmental validation showed Component 1 mask mean average precision at 50% intersection-over-union (mAP50) of 0.753 and box mAP50 of 0.883 and a Component 2 ICC of 0.792. Retuve can select analyzable frames and recover measurements from variable-quality cine sweeps, but alpha-angle calibration requires refinement. Future prospective work should evaluate developmental dysplasia of the hip (DDH) diagnostic accuracy, clinical treatment decision support, and screening outcomes.

## 1. Introduction

Developmental dysplasia of the hip (DDH) affects 1–3% of newborns and requires early detection to prevent long-term complications, including early-onset osteoarthritis [1]. Current screening relies on ultrasound examination, obtaining measurements in a standard plane first proposed by Graf [2]. With advances in probe technology, 2D cine sweep ultrasound now provides a dynamic assessment of hip morphology across multiple planes. While 2D sweep ultrasound offers valuable real-time visualization and is much easier to obtain by non-expert users than a single optimal Graf frame [3], the sweep approach inherently produces lower-quality images than static acquisitions due to motion artifacts and operator variability. Manual analysis of these dynamic cine sweep video sequences is time-consuming, operator-dependent, and lacks standardization [4].

Recent advances in deep learning have shown promise for automated DDH ultrasound analysis [5], but significant gaps remain in the literature. Several studies show that alpha angle, beta angle, or coverage can be estimated from a selected ultrasound frame using contour-based segmentation, landmark detection, or related measurement models [6,7,8,9,10]. However, single-frame measurement does not solve the upstream clinical problem of acquiring or identifying a standardized Graf plane. More recent systems, therefore, include standard-plane detection or video-based analysis, while 3D ultrasound methods attempt to recover hip morphology beyond a single 2D plane [11,12,13,14,15]. Image quality is also increasingly recognized as a determinant of reliable DDH assessment, with artificial intelligence (AI) approaches proposed for scan-quality classification and quality scoring [16,17,18,19]. Nevertheless, many studies still focus on static images without leveraging the dynamic nature of cine sweeps [20,21]. Others lack explicit scan quality considerations entirely, testing only on curated high-quality datasets [22]. Several works fail to include non-expert readers in validation, making human benchmarking difficult [11,23], while others provide unclear or absent documentation regarding image exclusion processes [19,24]. Prior Retuve validation was limited to single-frame public reference-quality images, and its ability to detect high-quality frames within cine sweep video had not yet been tested (https://github.com/radoss-org/retuve (accessed on 27 May 2026)). In the present study, we treat the Retuve framework as three dependent components: structure segmentation in each image frame, Graf-frame calibration, and final frame filtering with downstream measurements used in DDH assessment. Given the challenges of 2D sweep ultrasound analysis, automated systems must demonstrate strong end-to-end performance as well as robust performance within each component to ensure that each is sufficiently reliable to support the components that depend on it.

We performed this study to evaluate Retuve on a real-world dataset of 2D sweep ultrasound images of varying quality, focusing first on how well its three engineering components support one another and second on whether the resulting pipeline can recover alpha angle and coverage measurements on an optimal Graf frame despite inherent scan quality limitations. These measurements are central to DDH assessment, so agreement on alpha angle and coverage is an appropriate first target: a model could achieve good DDH classification accuracy while still showing poor agreement on the measurements themselves if it learned shortcuts or non-anatomical correlates rather than the intended anatomy. Component 3 depends on Components 1 and 2, because frame filtering and measurement cannot succeed without reliable segmentation and calibrated Graf-frame scoring. We therefore compare Retuve’s frame selection and measurements against expert radiologist annotations and assess interobserver reliability, while leaving direct diagnostic utility to future work.

## 2. Materials and Methods

### 2.1. Component Architecture and Dependencies

**Figure 1:**
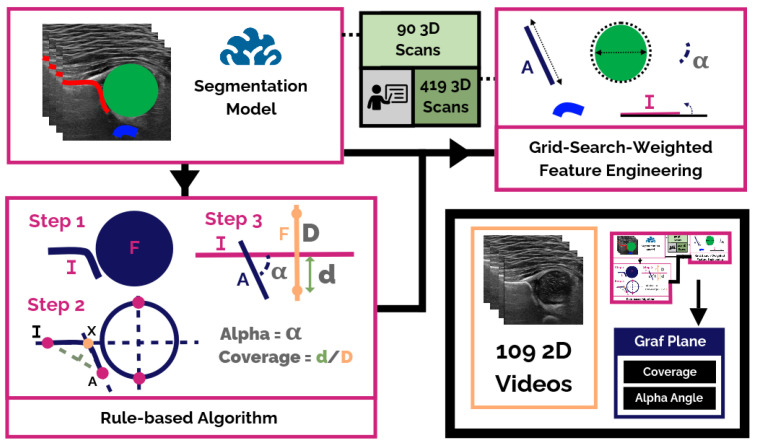
Three-component Retuve architecture: segmentation, Graf-frame calibration, and frame filtering with downstream contour-based measurements.

Retuve uses a contour-based pipeline: segmentation of key hip structures followed by deterministic rule-based engines for landmarks and Graf-frame measurements (Figure 1). The segmentation model was initialized from the default pretrained You Only Look Once version 11 (YOLOv11) weights and trained with ultrasound-specific augmentations (Table 1). No extensive hyperparameter tuning was performed beyond the default YOLO configuration. The contour algorithm skeletonizes the ilium segmentation and finds the apex point as the farthest distance from endpoints, enabling alpha angle and coverage calculations on any frame [5,25].

Retuve’s Graf-frame scoring algorithm uses seven continuous features: alpha angle, acetabular depth, femoral head size, femoral head roundness, ilium flatness, os ischium presence, and frame position within the acquisition sequence. These weighted features identify the optimal Graf frame, combining Graf’s methodological foundation with implementation details developed during prior Retuve work, American Institute of Ultrasound in Medicine–American College of Radiology–Society for Pediatric Radiology–Society of Radiologists in Ultrasound (AIUM-ACR-SPR-SRU) practice-parameter guidance, and local University of Alberta clinical practice [5,26,27,28]. Os ischium was included as an explicit segmentation class because its visibility contributes to both local and published criteria for assessing whether a hip ultrasound frame is suitable for DDH evaluation.

### 2.2. Component 1: Segmentation Training and Evaluation

This retrospective, multicenter study was conducted in three parts to evaluate Retuve for automated hip ultrasound frame selection and measurement against expert radiologist measurements. To ensure maximum generalizability, we used diverse datasets: Component 1 used 3D ultrasound from major hospitals in Philadelphia, USA; Component 2 used 3D ultrasound from a tertiary hospital in Alberta, Canada; and the final Component 3 evaluation used 2D sweep point-of-care ultrasound from a community primary care clinic in Alberta, Canada.

In Component 1, we used 3D ultrasound from 90 hips to train and evaluate YOLOv11 segmentation of the ilium/acetabular contour, femoral head, and os ischium using a 70:30 train/validation split. The 3D volumes were converted into 2D training images by extracting coronal orthogonal slices from the reconstructed volume, rather than using native 2D B-mode frames from the 3D probe. Pixel spacing metadata were checked so that frame and slice aspect ratios were preserved before model training. Training used the augmentation strategy shown in Table 2. Performance was summarized on the validation set using mean average precision (mAP) at intersection over union (IoU) 0.50 and mAP50-95, together with precision and recall, for both bounding boxes and masks. Nonparametric bootstrap 95% confidence intervals were estimated. Because no locked held-out test set was maintained for Component 1, these metrics are reported as internal developmental validation and may be optimistic relative to performance on an independent external segmentation test set.

### 2.3. Component 2

For Component 2, we calibrated Graf-frame scoring using Component 1-derived contours on 419 3D ultrasound scans with semi-expert labels from Alberta, Canada, using a 90:10 train/validation split; therefore, the reported Component 2 intraclass correlation coefficient (ICC) reflects the upstream segmentation-to-calibration chain rather than isolated calibration performance. This evaluation was performed on 3D data because those volumes provided more controlled anatomical coverage for tuning the seven handcrafted Graf-frame features before their transfer to noisier 2D cine sweeps. For each frame, the calibration score was defined as

(1) $$S=\sum_{i=1}^{7}w_{i}x_{i}=5.6x_{1}+0.96x_{2}+12.81x_{3}+1.42x_{4}+4.71x_{5}+14.51x_{6}+0.16x_{7},$$

where the seven continuous features $x_{i}$ correspond to the alpha angle, acetabular depth, femoral head size, femoral head roundness, ilium flatness, os ischium presence, and frame position in the sequence. The final term was used only for 3D scans, so the frame-position feature was disabled for non-3D acquisitions. The algorithm cycles through all frames in a scan and selects the frame with the highest score. We evaluated 2100 randomly sampled candidate weight combinations. In each candidate combination, all seven weights were independently sampled between 0 and 15 and rounded to a maximum of two decimal places. This sampling count was chosen arbitrarily as a practical exploratory calibration strategy rather than as a formal ablation or optimization study. For each sampled weight set, the average ICC loss was computed against semi-expert Graf-frame positions across the validation subset, and the weight set with the lowest ICC loss was selected. The sampled distribution of ICC loss values was used descriptively as a sensitivity analysis for the weighting scheme rather than as a parametric statistical test; extreme, failed candidate weight sets with ICC loss >0.6 were excluded from the descriptive mean and SD, representing less than 1.5% of the sampled combinations. No locked held-out test set was maintained for Component 2, so this calibration result is reported as internal developmental validation rather than an unbiased external performance estimate. Calibration also defined transparent rule-based robustness filters: plausibility limits for the alpha angle and coverage, geometry checks on acetabular span and ilium flatness, connected-component artifact removal, sliding-window outlier rejection, femoral head area and roundness thresholds, and multi-ilium handling rules. Calibration stability and the final selected weights were summarized using the distribution of ICC loss values and the final ICC against the semi-expert labels.

### 2.4. Component 3: External Test Set on 2D Sweep Ultrasound

Component 3 externally evaluated the integrated Component 1-to-Component 2-to-Component 3 pipeline on 2D sweep ultrasound videos from an independent community primary care clinic in Alberta (109 hips). This dataset was entirely distinct from both the Philadelphia Component 1 data and the Alberta 3D Component 2 data. Although Components 2 and 3 were both collected in Alberta, they differed in source site, acquisition format, and labeled task. Pixel spacing and image aspect ratios were checked during preprocessing, but depth settings, probe frequencies, gain, and acquisition presets were not identical across centers or acquisition formats. We therefore treated this as an expected domain shift between tertiary-center 3D ultrasound and community-clinic 2D point-of-care ultrasound (POCUS) cine sweeps; the training augmentations in Table 1 were intended to improve robustness to such differences in noise, contrast, blur, and apparent resolution. No scans were excluded based on quality criteria before analysis. Each cine sweep video contained over 300 frames, totaling over 32,000 frames across the analyzed examinations. Less than 5% of frames (107 expert-labeled Graf frames) were identified as analyzable by human experts for clinical measurements. Although the number of videos was modest, the test required frame selection within a large uncurated search space. The algorithm used the seven features described in Section 2.1 to score each frame, select the best candidate Graf frame, and then pass accepted frames to the contour-based alpha-angle and coverage measurement pipeline. No algorithm training or tuning was performed on this external test set. Because prior Retuve validation had been limited to single-frame reference-quality images, this component specifically tested whether quality assessment and downstream measurement remained reliable in uncurated cine sweeps.

**Figure 2:**
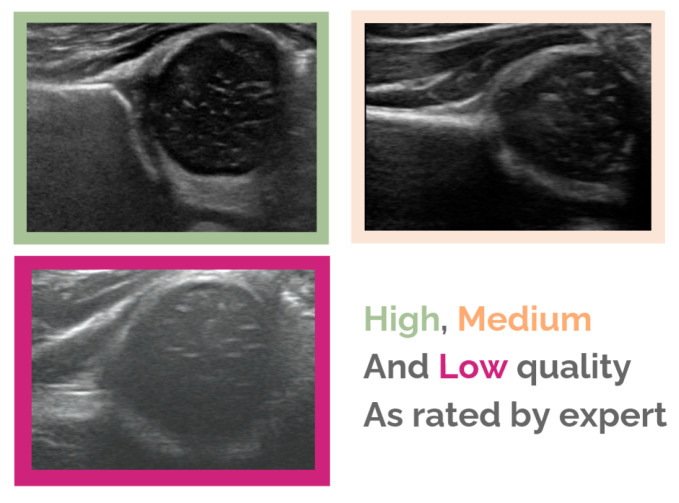
Examples of high-, medium-, and low-quality scans on the 10-point scoring system [18], illustrating how POCUS data quality varies across the datasets. The high-quality example is adapted from Radiopaedia [29]; the remaining examples are from the Canadian study centres.

**Figure 3:**
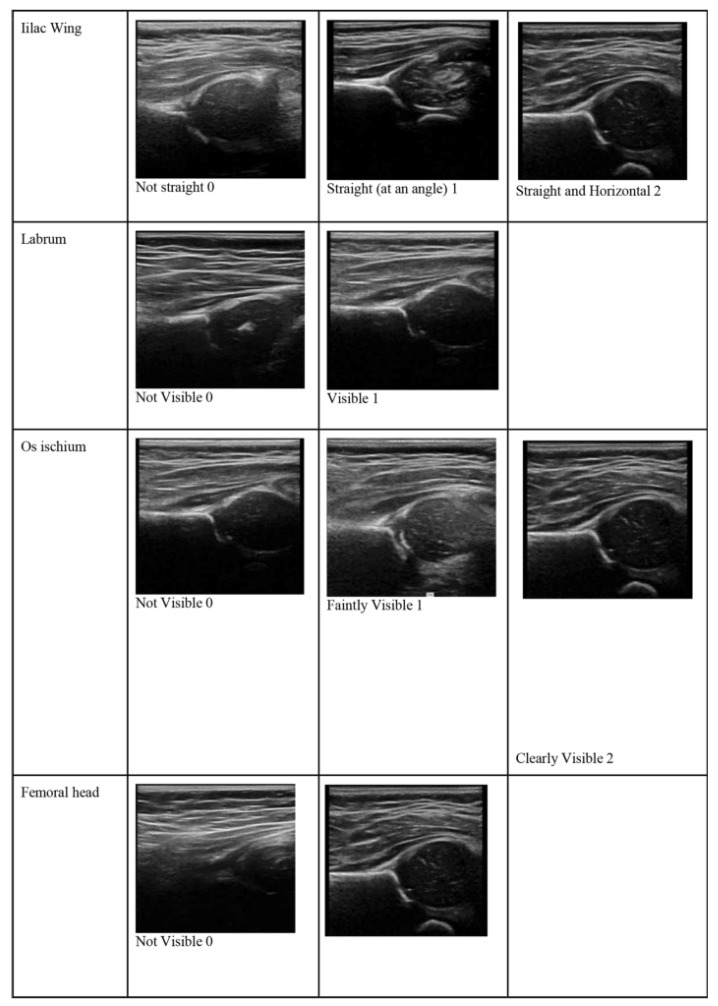
Definition of the 10-point scan quality scoring system from the referenced paper [18]: ilium (0–2), labrum (0–1), os ischium (0–2), femoral head (0–1), motion artifact (0–2), and other imaging artifacts such as limited penetration or excessive image noise (0–2).

**Figure 4:**
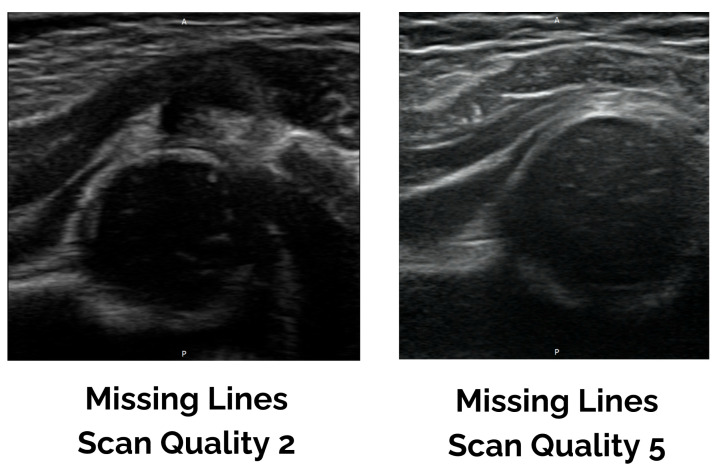
Best Graf-quality frames from the two excluded scans (**left**: Scan A, **right**: Scan B). They have expert-labeled scan quality values, but both were missing annotation lines required for calculating coverage and alpha angle.

We show example images at different points on the scan quality scale from our datasets in Figure 2. The expert radiologist additionally provided scan quality ratings on a 10-point scale to characterize the test data [18]; Figure 3 shows the scoring system definition. Due to a bug in the labeling software, 2 scans were excluded from frame-selection analysis due to missing labels, leaving 107 scans for comprehensive frame-selection evaluation. As shown in Figure 4, the exclusions were due to technical data integrity issues with the labeling software rather than image quality. One expert dual-fellowship-trained pediatric musculoskeletal radiologist (15+ years of experience in pediatric hip ultrasound) and one radiology fellow provided reference standard annotations for alpha angle and coverage measurements on a selected optimal Graf frame from each 2D sweep sequence. All external test set annotations were performed independently without knowledge of AI predictions.

For quality assessment, the unit of analysis was the whole-sweep video: we assessed whether the expert and AI agreed that at least one analyzable frame existed anywhere within the sweep. For alpha angle and coverage, the unit of analysis was the selected frame, and agreement was quantified on the continuous measurements themselves using ICC (absolute and consistency), comparing AI, expert radiologist, and fellow with a two-way mixed-effects, single-measure model for consistency, reported as ICC (3, 1) [30]. We prioritized measurement agreement because alpha angle and coverage are the quantities used in DDH assessment; agreement on these metrics provides evidence that the model is following clinically meaningful anatomy rather than only learning features that separate classes. Secondary assessment included over-marking and under-marking counts, confusion-matrix analysis, and case-by-case AI failure review.

Over-marking refers to instances where the AI analyzed a case that the expert radiologist excluded as non-diagnostic or poor quality, while under-marking refers to instances where the AI failed to analyze a case that the expert deemed suitable for measurement. We generated a confusion matrix to compare expert opinion against AI determination as to whether a scan was of adequate quality to be analyzable.

Thus, under-marked cases correspond to false negatives (FNs) and represent examinations the AI failed to identify as containing any analyzable frame when the expert did, while over-marked cases correspond to false positives (FPs) and represent examinations the AI analyzed when the expert excluded them as non-analyzable. Uncertainty for frame-selection sensitivity and specificity was summarized using Wilson 95% confidence intervals. Measurement bias was evaluated using scatter plot analysis, ICC comparisons, and Bland–Altman analysis to identify systematic differences between Retuve and human annotations.

## 3. Results

### 3.1. Component 3: External Test of Frame Filtering and Downstream Measurement

**Figure 5:**
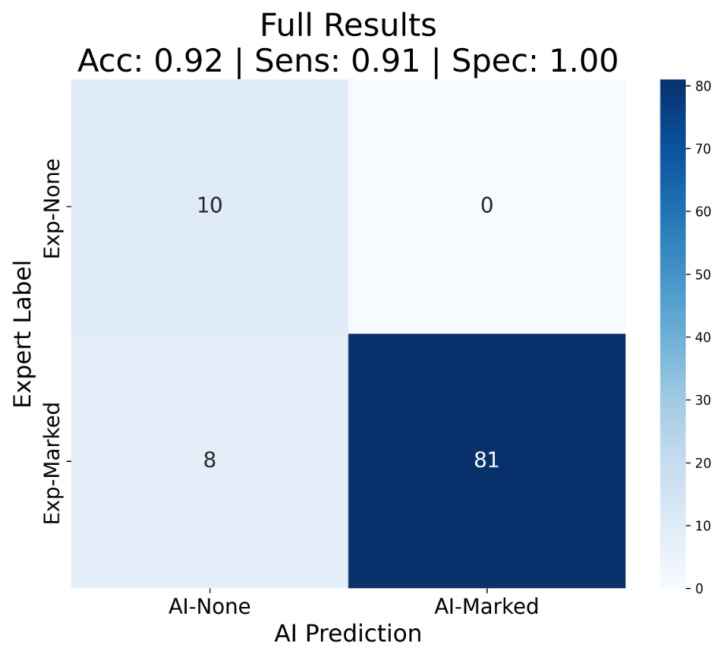
Confusion matrix for exam-level frame-assessment performance. The AI correctly identified 91% of examinations with at least one analyzable frame (95% CI 83.6–95.5) and 100% of examinations without any analyzable frame (95% CI 80.6–100.0).

**Figure 6:**
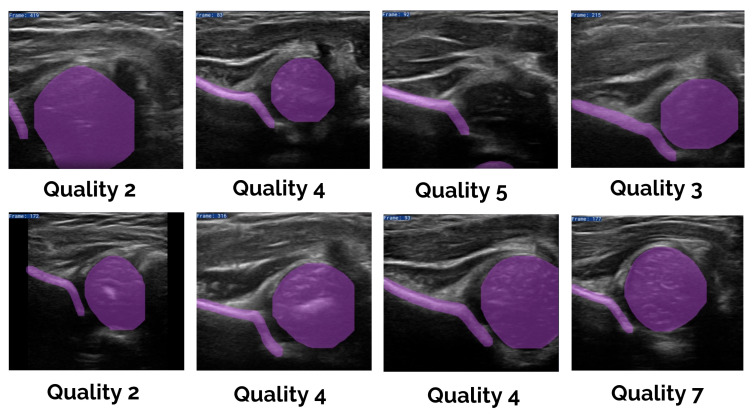
The eight under-marked cases. The frame shown in each panel is the expert-selected best frame from that cine sweep. One case (top left) shows the absence of the ilium, whereas the other seven cases show a non-flat ilium. The bottom-right case had a scan quality score of 7/10 despite a similarly non-flat ilium, likely because a visible os ischium contributes additional points to the additive quality score.

**Figure 7:**
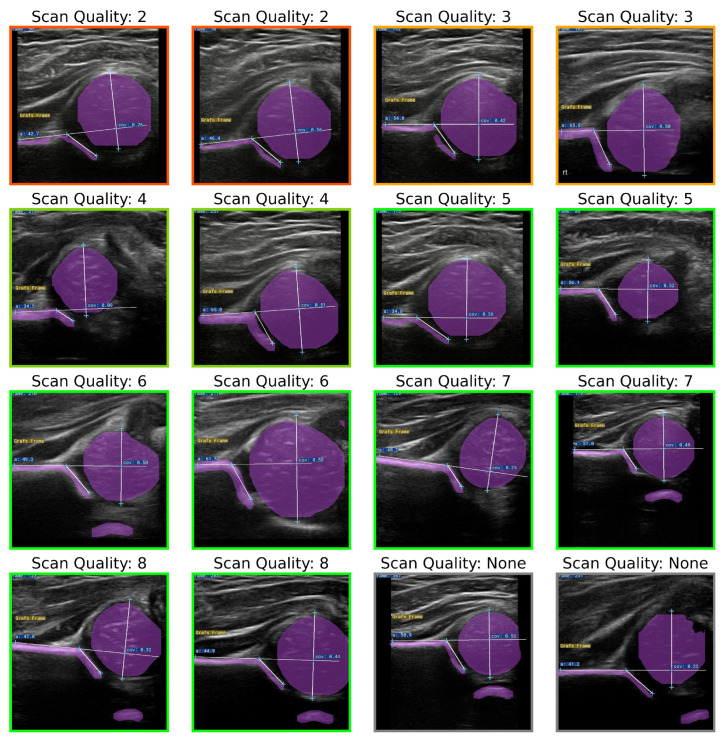
Representative examples from the Component 3 external test set across different scan qualities. In general, Retuve successfully selected frames when the key landmarks required for Graf-based analysis were visible, particularly a flat ilium together with the femoral head. In more ambiguous or lower-quality cases, the model still made best-effort selections that were broadly consistent with expert judgment.

The final frame-selection analysis included 107 2D sweep ultrasound examinations, reflecting challenging, real-world POCUS quality, with an average expert image quality score of just 4.4/10 [18]. By expert assessment, 91 examinations contained at least one analyzable frame, and 16 contained no analyzable frame. At the whole-sweep level, the AI achieved 100% specificity (95% CI 80.6–100.0) and 91% sensitivity (95% CI 83.6–95.5) for agreement with the expert on this frame-availability task, corresponding to 83 true positives, 8 false negatives, 16 true negatives, and 0 false positives. Because specificity was estimated from only 16 expert-negative examinations, the confidence interval is more informative than the point estimate alone. The radiology fellow achieved 75% specificity (95% CI 50.5–89.8) and 96% sensitivity (95% CI 89.2–98.3) on the same task (Table 3; Figure 5). The AI therefore behaved more conservatively, with 8 under-marked cases and 0 over-marked cases. Review of these 8 cases (Figure 6) showed that 1 case lacked a visible ilium, while the remaining 7 demonstrated a non-flat ilium despite being the expert-selected best frame for that cine sweep. Figure 7 provides representative examples from the Component 3 external test set across a range of scan qualities. In general, when a frame contained the key landmarks needed for Graf-based analysis, particularly a flat ilium together with a visible, large femoral head, Retuve selected it. In the remaining borderline cases, the model made best-effort selections that were broadly aligned with expert behavior in the setting of limited image quality.

Interobserver agreement between the expert radiologist and the radiology fellow established the human benchmark. For alpha angle measurements, ICC values were 0.69 (absolute, 95% CI 0.56–0.79) and 0.70 (consistency, 95% CI 0.59–0.79). Coverage measurements showed ICC values of 0.78 for both absolute and consistency agreement (95% CI 0.69–0.85) [30].

**Figure 8:**
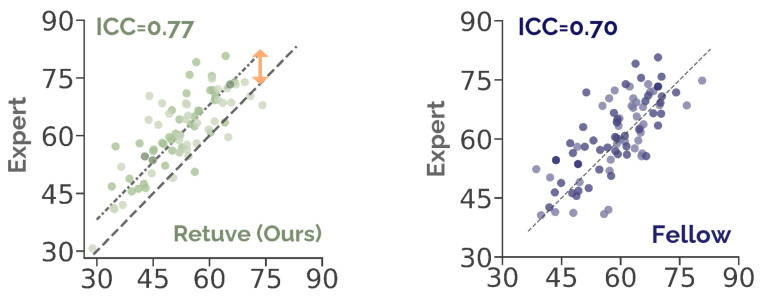
Consistency ICC for alpha angle: Retuve (0.67–0.84) vs. Radiology Fellow (0.59–0.79). Transparency represents scan quality as rated by the expert Radiologist—less transparent means higher scan quality. The Orange Arrow represents the bias measured from Retuve relative to the expert.

**Figure 9:**
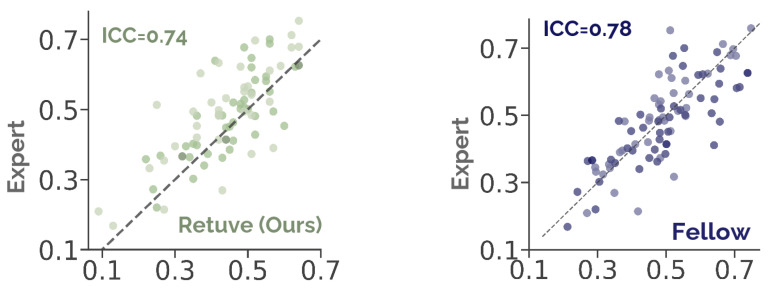
Consistency ICC for coverage: Retuve (0.63–0.82) vs. Radiology Fellow (0.69–0.85).

Compared to the expert radiologist, Retuve achieved alpha-angle ICC values of 0.55 for absolute agreement (95% CI −0.07–0.81) and 0.77 for consistency agreement (95% CI 0.67–0.84). The lower absolute-agreement ICC and negative lower confidence bound indicate systematic measurement bias despite preserved rank/linear consistency. Bland–Altman analysis confirmed this alpha-angle bias: Retuve overestimated the alpha angle by 8.53° on average, compared with 1.99° for the radiology fellow (Appendix C). For coverage, absolute and consistency ICC values were both 0.74 (95% CI 0.63–0.82 and 0.62–0.82, respectively) [30]. These results reflect agreement on continuous alpha angle and coverage measurements once a frame had been selected for comparison. The measured sample had a mean alpha angle of 68 ± 7 and a mean coverage of 0.6 ± 0.11, suggesting that the randomly sampled validation cohort was skewed toward normal hips. Future diagnostic studies should include larger numbers of frankly dysplastic and borderline hips. Comparisons for alpha angle and coverage are shown in Figure 8 and Figure 9.

### 3.2. Internal Validation of Upstream Components

#### 3.2.1. Component 1: Segmentation Performance

**Figure 10:**
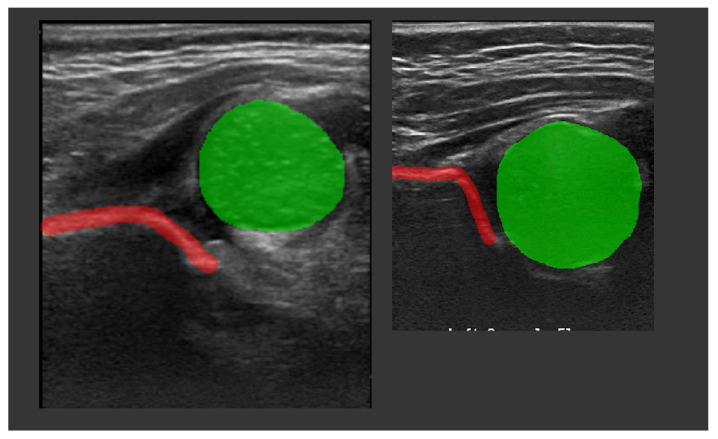
Segmentation quality analysis showing how probe-setting differences can produce different apparent ilium thicknesses (red line width) despite consistent annotations, potentially affecting skeletonization and downstream measurements. Green circle represents the Femoral Head Segmentation.

In internal developmental validation, the segmentation model achieved a pooled box mAP50 of 0.883 and a pooled mask mAP50 of 0.753 (Table 4). These values describe validation-fold behavior during upstream model development, not locked-test performance. Box detection was strongest for the ilium/acetabular contour (AP50 0.991, 95% CI 0.989–0.993), whereas mask AP50 for this same class was lower (0.608, 95% CI 0.572–0.652). Femoral head and os ischium performance was similar for boxes and masks, suggesting that when these structures were detected, their masks were generally accurate. The os ischium had lower mAP50-95 than AP50, indicating some sensitivity to stricter mask-overlap thresholds; this is less important for the present Graf-frame selection task, where os ischium presence is used mainly as a quality feature, but would matter more for future models that calculate pubofemoral distance [31]. The lower ilium/acetabular mask performance likely reflects the difficulty of obtaining consistent pixel-level boundary annotations in lower-quality ultrasound rather than a failure of gross structure detection. Figure 10 shows a likely contributor: probe-setting differences can change the apparent thickness of the ilium, so even consistent annotations may produce skeletonization differences at the boundary level. Full per-class precision, recall, AP50, mAP50-95 values, and precision–recall curves are provided in Appendix A.

#### 3.2.2. Component 2: Graf-Frame Calibration

**Figure A3:**
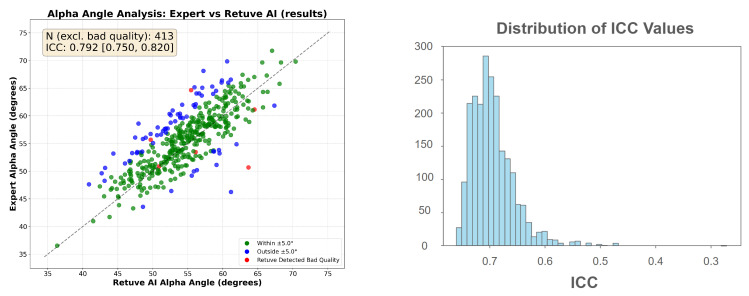
Calibration analysis: (**left**) Alpha-angle ICC performance for Retuve and the radiology fellow against expert measurements. (**right**) Graf-frame calibration performance against semi-expert labels and sensitivity of ICC loss across 2100 randomly sampled candidate weight combinations.

In internal developmental validation, calibration on 419 3D ultrasound scans showed stable parameter behavior across 2100 randomly sampled candidate weight combinations. After excluding extreme failed candidate weight sets with ICC loss >0.6 from the descriptive summary, representing less than 1.5% of sampled combinations, the mean ICC loss was 0.3 (SD 0.05). The final selected weighting set achieved an ICC of 0.792 against semi-expert Graf-frame labels (loss 0.208), as shown in Figure A3. This value documents the validation-fold behavior of the segmentation-to-calibration chain rather than an independent external estimate.

## 4. Discussion

This study evaluates the performance of the Retuve AI analysis tool on hip ultrasound 2D sweep videos, considering it as a sequence of three dependent engineering components. An important contribution is the direct frame-selection evaluation in cine sweeps, which had not previously been evaluated for Retuve. In this cohort, Retuve showed higher exam-level specificity than the radiology fellow (100% [95% CI 80.6–100.0] vs. 75% [95% CI 50.5–89.8]) for agreement with the expert on whether any analyzable Graf frame was present. Unlike analyses performed only on pre-selected standard planes, this evaluation addresses the engineering problem of identifying candidate measurement frames within uncurated cine sweeps.

The measurement results (consistency ICC of 0.77 for alpha angle and 0.74 for coverage) indicate that once analyzable frames are isolated, the contour-based measurement component can achieve consistency comparable to the human benchmark in this cohort. This consistency should be interpreted alongside the lower alpha-angle absolute-agreement ICC (0.55, 95% CI −0.07–0.81) and Bland–Altman bias (+8.53°), which indicate systematic alpha-angle overestimation and a need for further calibration before Retuve measurements are used as substitutes for expert measurements. We focused on metric agreement because alpha angle and coverage are the measurements used to quantify hip morphology in DDH assessment. A model could, in principle, produce acceptable DDH classification while still correlating poorly with these measurements, which would raise concern that it had learned non-causal visual shortcuts rather than the intended anatomical relationships. On this basis, metric agreement is an appropriate development target that can support future work on direct DDH classification and prospective diagnostic utility.

### 4.1. Scan Quality and Comparisons with Literature

As expected in our dataset, which deliberately included a wide range of low-quality scans, agreement between the AI and human expert was generally lower than in prior studies that evaluated more curated or higher-quality data [12]. Retuve also performed below its previously reported results on the higher-quality Open Hip Dataset (consistency ICC of 0.86 for alpha angle and 0.92 for coverage) [5,29,32,33,34,35,36,37,38,39,40,41,42,43,44,45,46].

A decline in performance on community-acquired data in this study vs. prior performance on high-quality 3D ultrasound data is expected and was, fortunately, relatively small. Retuve was tested on real-world data through a component-based architecture in which each component supports the next. The 100% specificity in frame filtering (95% CI 80.6–100.0) should be interpreted cautiously because it is based on only 16 true-negative examinations and reflects expert-AI agreement on exam-level analyzability. The accompanying 91% sensitivity (95% CI 83.6–95.5) quantifies the tradeoff of this conservative quality assessment behavior. In practice, maintaining good downstream agreement on alpha angle and coverage requires rejecting many candidate frames so that only geometrically plausible Graf frames are passed to the measurement stage. The measurement agreement on the isolated frames (ICC 0.77–0.74) indicates that this assessment step can be followed by quantitative measurement with agreement comparable to the human benchmark in this cohort, but broader testing is needed in cohorts enriched for dysplastic and borderline hips.

If future studies evaluate Retuve within POCUS-based hip dysplasia screening workflows, variable scan quality will be a central challenge; therefore, the evaluation should include all levels of image quality rather than curated standard planes alone.

Direct comparison with prior DDH ultrasound AI studies is therefore limited. Several published works analyze pre-selected standard planes [12], exclude poor-quality scans [8], or evaluate datasets with higher average image quality [9]. The present study differs by testing frame selection and downstream measurement in noisy, uncurated cine sweeps rather than only in pre-selected images.

The under-marked cases also suggest an important limitation in the 10-point scan quality score used to contextualize performance (Figure 3 and Figure 6). In the referenced additive scoring system, the presence of the os ischium increases the total score. In practice, however, a visible os ischium may co-occur with a non-flat ilium and therefore can be misleading when interpreted as a linear positive contributor to overall Graf-frame suitability. This may explain why the bottom-right under-marked case still received a quality score of 7/10 despite a similarly non-flat ilium. For future scan quality models, the os ischium should likely be treated as a penalty or conditional feature, rather than as a feature that always increases quality, because its current additive treatment may too easily reward frames with suboptimal iliac morphology.

### 4.2. Value of Low-Quality Scans in AI Training and Validation

Including low-quality scans was a deliberate design choice rather than a limitation. In POCUS practice, motion artifact, suboptimal positioning, and operator variability are common, so an AI system intended for clinical use must learn to distinguish analyzable from non-analyzable frames rather than relying on curated data alone. These difficult cases function as hard negatives, which is consistent with the observed exam-level specificity in frame selection (100%, 95% CI 80.6–100.0), although that estimate is based on only 16 true-negative examinations. The agreement achieved on isolated valid frames (ICC of 0.77 for alpha angle and 0.74 for coverage) further suggests that the pipeline can recover useful measurements from noisy acquisitions.

### 4.3. Systematic Bias

**Figure 11:**
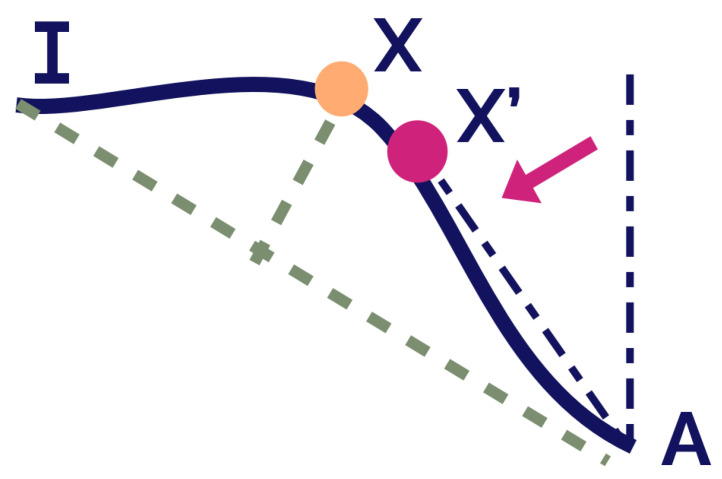
Apex-point selection: Retuve (farthest distance from line IA, which is X) vs. Graf method (tangent to acetabular roof, X’).

The systematic bias in alpha angle measurements (Retuve tending toward higher values) is reflected by the lower alpha-angle absolute-agreement ICC (0.55, 95% CI −0.07–0.81) despite a higher consistency ICC (0.77, 95% CI 0.67–0.84) and by the Bland–Altman alpha-angle bias of +8.53° (Appendix C). This may reflect differences in apex-point selection: Retuve uses an orthogonal-distance algorithm robust to endpoint variations, while Graf’s method relies on the deepest acetabular point, which can vary (Figure 11) [26,27]. This bias limits the interpretation of the alpha-angle measurement as an absolute replacement for expert measurement without further expert-calibrated adjustment.

### 4.4. Cross-Modality Generalization

Retuve was evaluated across centers and acquisition formats in this study. Unlike landmark-based approaches, the contour-based method learns global hip geometry, enabling feature extraction from noisy 2D POCUS inputs despite temporal and spatial variability [5,6]. This cross-format evaluation should be interpreted in light of known acquisition differences: Components 1 and 2 used coronal slices from 3D ultrasound volumes, whereas Component 3 used native 2D cine sweeps acquired in a community clinic. Pixel spacing and aspect ratios were checked, but probe frequencies and imaging presets were not harmonized across centers. These findings therefore suggest that 3D-derived training data provide anatomical representations that remain usable in a challenging real-world domain, although broader testing across ultrasound systems, probe frequencies, and acquisition presets would be needed to establish generalization.

### 4.5. Deterministic Rule Engine vs. Black Box: Clinical and Technical Advantages

Our hybrid approach combines YOLOv11n segmentation with deterministic rule-based measurements. The contour-based measurement algorithm operates on explicit *if-then* logic (plausibility limits: alpha [0°,90°], coverage [0.0,0.9]), providing auditable decision paths that contrast with opaque black-box models [47,48]. Rule engines handle missing data explicitly [49], and in this cohort, the system’s robustness filters correctly rejected all 16 expert-defined non-analyzable examinations. This conservative filtering behavior likely helps preserve downstream measurement agreement.

### 4.6. Open Source Data

The field lacks standardized, publicly available DDH ultrasound datasets across modalities and quality levels, hindering model comparison and clinical translation.

### 4.7. Limitations and Future Direction

This study had several limitations, including: (1) the need for future Component 3 cohorts with confirmed DDH diagnosis to evaluate diagnostic accuracy against an external reference standard, treatment decision performance, and screening effectiveness; (2) retrospective study design with 107 external test videos for final frame-selection analysis, including only 16 expert-negative non-analyzable examinations for estimating specificity; (3) likely dataset bias toward normal cases, with mean alpha angle 68 ± 7, mean coverage 0.6 ± 0.11, and unknown prevalence of confirmed DDH in the 2D sweep cohort, limiting confidence in performance for dysplastic or borderline hips; (4) the 2D sweep external test cohort was obtained from a single community clinic, even though the overall study was multicenter across Components 1–3 (reflecting a field-wide scarcity of saved POCUS cine sweeps); (5) no locked held-out test sets were maintained for Components 1 and 2; (6) potential variability across ultrasound systems, probe frequencies, imaging presets, and operators; (7) possible annotation methodology differences between calibration and test phases; (8) dependence on expert annotations from our Canadian center, which may reflect local interpretive preferences that differ from those of experts in other institutions and countries; and (9) the Component 2 weighting scheme was calibrated using an arbitrary random-sampling strategy rather than a full ablation study of the scan-quality features.

Manual frame evaluation remains bottlenecked by the need to screen over 300 frames per exam for the <5% optimal Graf frames. Future work should evaluate diagnostic accuracy and workflow efficiency prospectively, test direct DDH classification once measurement validity and frame selection have been established more broadly, and perform full ablation studies of scan-quality feature weighting for hip sonography.

## 5. Conclusions

Retuve, an open-source automated AI tool for hip ultrasound image assessment, passed challenging tests of each of its three components in this study. It was able to select high-quality image frames from cine sweep ultrasound videos and calculated alpha angle and coverage measurements, with measurement consistency comparable to the human benchmark in this cohort. The observed alpha-angle absolute-agreement bias indicates that further calibration is needed before Retuve measurements are used as substitutes for expert measurements. The results of this study support combining frame selection and quantitative measurement as a sequential pipeline for ultrasound image analysis. Future prospective studies are needed to assess Retuve’s diagnostic accuracy in hip dysplasia and to determine whether this pipeline can safely support screening workflows involving non-expert users.

## Figures and Tables

**Table 1:** Ultrasound-specific image augmentations used during training; *p*, probability of applying the augmentation.

Augmentation	Parameters
Multiplicative Noise	multiplier (0.95, 1.05); *p* = 0.3
Gauss Noise	var_limit (5.0, 15.0); *p* = 0.25
Blur/Motion Blur	blur_limit 3; *p* = 0.35
Brightness/Contrast/Gamma	limits 0.1–0.15; *p* = 0.3
Downscale	scale_min 0.25; *p* = 0.5

**Table 2:** Study datasets and analysis units across the three components.

Comp.	Source	Modality	Scans/Videos	Split	Task	Main Analysis Unit
1	Philadelphia tertiary hospital, USA	3D US	90 hips	70:30 train/val	Segmentation of ilium/acetabular contour, femoral head, and os ischium	Hip for validation metrics (mAP, precision, recall), reported by class and pooled across structures
2	Alberta tertiary center, Canada	3D US	419 hips	90:10 train/val	Graf-frame calibration from seven continuous features	Hip, with agreement to semi-expert frame selection summarized by ICC on validation
3	Alberta primary care clinic, Canada	2D US cine	109 videos; 107 for quality assessment	External test	Detection of at least one analyzable Graf frame, plus alpha angle and coverage on one selected frame	Video for quality assessment; selected frame for alpha angle and coverage ICC

Abbreviations: US, ultrasound; val, validation; mAP, mean average precision; ICC, intraclass correlation coefficient.

**Table 3:** Frame-selection performance comparison for identifying examinations that did or did not contain at least one analyzable frame.

Method	TP	FN	TN	FP	Specificity (95% CI)	Sensitivity (95% CI)
AI Algorithm	83	8	16	0	100% (80.6–100.0)	91% (83.6–95.5)
Radiology Fellow	87	4	12	4	75% (50.5–89.8)	96% (89.2–98.3)

**Table 4:** Key Component 1 internal validation segmentation metrics. Values are mean (95% bootstrap CI). Full precision, recall, AP50, and mAP50-95 results are provided in Appendix A.

Class	Box AP50	Mask AP50	Box mAP50-95	Mask mAP50-95
Ilium/acetabulum	0.991 (0.989–0.993)	0.608 (0.572–0.652)	0.727 (0.714–0.740)	0.264 (0.245–0.283)
Femoral head	0.853 (0.822–0.875)	0.851 (0.818–0.872)	0.658 (0.628–0.679)	0.643 (0.613–0.665)
Os ischium	0.806 (0.767–0.848)	0.801 (0.761–0.846)	0.521 (0.490–0.550)	0.388 (0.362–0.412)
**Pooled**	**0.883 (0.767–0.993)**	**0.753 (0.572–0.872)**	**0.635 (0.490–0.740)**	**0.432 (0.245–0.665)**

**Figure A1:**
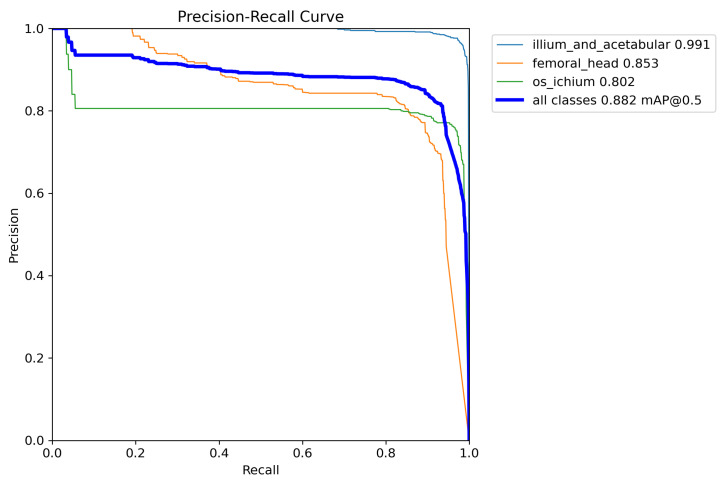
Component 1 bounding-box precision–recall curves for the internal validation set.

**Figure A2:**
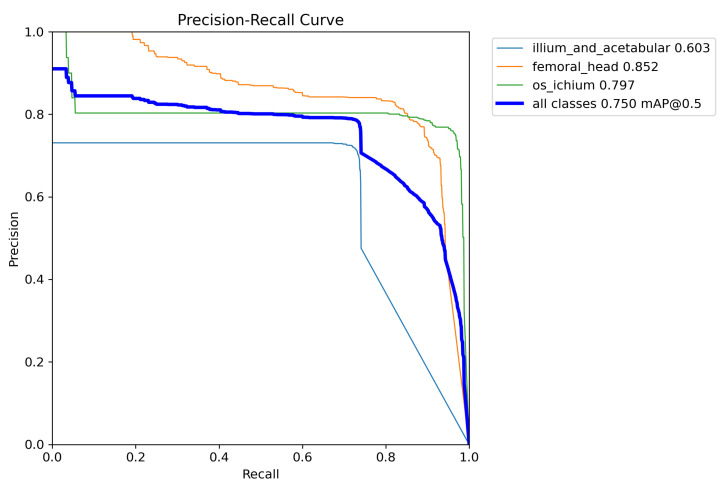
Component 1 mask precision–recall curves for the internal validation set.

**Figure A4:**
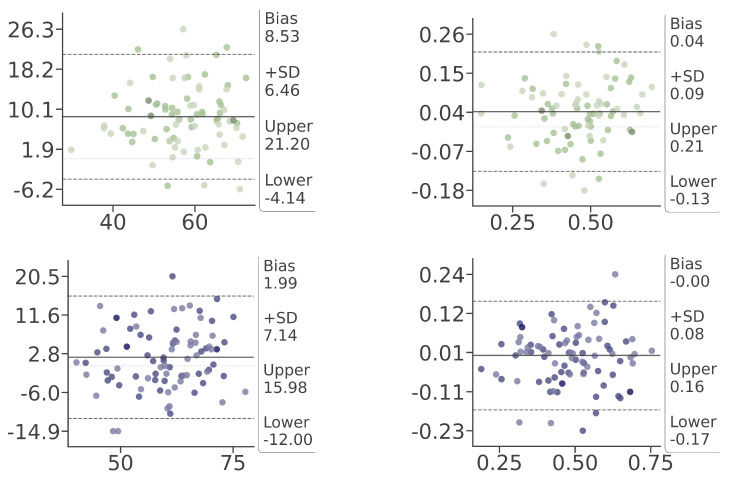
Bland–Altman plots for Component 3 measurement agreement against the expert radiologist: Going left to right, Retuve alpha angle and coverage (**green**), radiology fellow alpha angle and radiology fellow coverage (**blue**). Summary statistics are provided in Table A3.

## Data Availability

The data supporting the reported results are available upon request and subject to a data sharing agreement. Interested researchers may contact Jacob L. Jaremko at jjaremko@ualberta.ca to discuss the terms of access.

## Abbreviations

The following abbreviations are used in this manuscript:

AI	Artificial Intelligence
AIUM-ACR-SPR-SRU	American Institute of Ultrasound in Medicine–American College of Radiology–Society for Pediatric Radiology–Society of Radiologists in Ultrasound
AP50	Average Precision at 50% Intersection over Union
CI	Confidence Interval
DDH	Developmental Dysplasia of the Hip
FN	False Negative
FP	False Positive
ICC	Intraclass Correlation Coefficient
IoU	Intersection over Union
LOA	Limits of Agreement
mAP	Mean Average Precision
mAP50	Mean Average Precision at 50% Intersection over Union
POCUS	Point-of-Care Ultrasound
SD	Standard Deviation
TN	True Negative
TP	True Positive
US	Ultrasound
val	Validation
YOLOv11	You Only Look Once version 11

## Appendix A. Component 1 Segmentation Results

**Table A1:** Full Component 1 internal validation segmentation metrics. Values are mean (95% bootstrap CI) from 200 bootstrap iterations.

Metric	Output	Ilium/Acetabulum	Femoral Head	Os Ischium	Pooled
Precision	Box	0.957 (0.934–0.973)	0.751 (0.705–0.789)	0.788 (0.745–0.824)	0.832 (0.705–0.973)
Precision	Mask	0.713 (0.682–0.751)	0.747 (0.697–0.787)	0.784 (0.737–0.820)	0.748 (0.682–0.820)
Recall	Box	0.985 (0.974–0.994)	0.898 (0.871–0.921)	0.903 (0.856–0.959)	0.929 (0.856–0.994)
Recall	Mask	0.735 (0.708–0.768)	0.897 (0.872–0.919)	0.907 (0.857–0.967)	0.846 (0.708–0.967)
AP50	Box	0.991 (0.989–0.993)	0.853 (0.822–0.875)	0.806 (0.767–0.848)	0.883 (0.767–0.993)
AP50	Mask	0.608 (0.572–0.652)	0.851 (0.818–0.872)	0.801 (0.761–0.846)	0.753 (0.572–0.872)
mAP50-95	Box	0.727 (0.714–0.740)	0.658 (0.628–0.679)	0.521 (0.490–0.550)	0.635 (0.490–0.740)
mAP50-95	Mask	0.264 (0.245–0.283)	0.643 (0.613–0.665)	0.388 (0.362–0.412)	0.432 (0.245–0.665)

## Appendix B. Component 2 Calibration Results

**Table A2:** Component 2 calibration sensitivity summary across sampled weight sets.

Quantity	Value or Interpretation
Sampled weight sets	2100 randomly sampled candidate combinations of the seven feature weights
Objective	Average ICC loss against semi-expert Graf-frame labels
Use of sampled distribution	Descriptive sensitivity analysis of calibration stability, not a parametric statistical test
Extreme failed candidate rule	ICC loss >0.6 excluded from descriptive mean and SD
Extreme failed candidates	<1.5% of sampled candidate combinations
Descriptive ICC loss after exclusion	Mean 0.3; SD 0.05
Selected weight-set performance	ICC 0.792 against semi-expert labels; ICC loss 0.208

## Appendix C. Component 3 Bland–Altman Analysis

**Table A3:** Bland–Altman summary for Component 3 alpha-angle and acetabular coverage measurements against the expert radiologist.

Comparison	Measurement	Bias	SD	Lower LOA	Upper LOA	N
Retuve vs. expert	Alpha angle (°)	8.53	6.46	−4.14	21.20	81
Retuve vs. expert	Coverage	0.0433	0.0864	−0.1260	0.2125	81
Fellow vs. expert	Alpha angle (°)	1.99	7.14	−12.00	15.98	93
Fellow vs. expert	Coverage	−0.0030	0.0833	−0.1663	0.1603	93
